# *N*-(4-Methyl­phen­yl)-*N*-{[(2-nitro­phen­yl)acet­yl]­oxy}benzamide

**DOI:** 10.1107/S1600536812039360

**Published:** 2012-09-22

**Authors:** Dong-Hui Qu, Hong-Xia Zhang, Jing Ma

**Affiliations:** a Gansu Health Center Hospital, Lanzhou 730000, Gansu Province, People’s Republic of China; b Institute of Medicinal Chemistry, School of Pharmacy, Lanzhou University, Lanzhou 730000, Gansu Province, People’s Republic of China

## Abstract

In the title mol­ecule, C_22_H_18_N_2_O_5_, the nitro-substituted benzene ring makes dihedral angles of 71.56 (1)° with the benzoyl ring and 16.28 (1)° with the methyl-substituted benzene ring. The crystal structure features C—H⋯O inter­actions, which generate chains.

## Related literature

For biological applications of hydroxamic acid derivatives, see: Noh *et al.* (2009 ▶); Zeng *et al.* (2003 ▶). For the preparation of the title compound, see: Ayyangark *et al.* (1986 ▶).
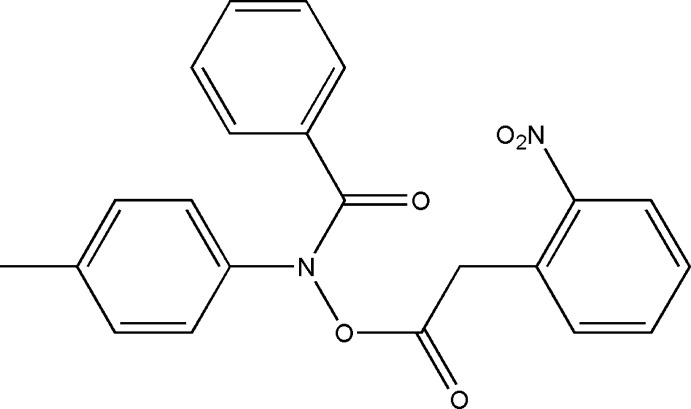


## Experimental

### 

#### Crystal data

C_22_H_18_N_2_O_5_
*M* *_r_* = 390.38
Orthorhombic, 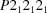

*a* = 9.246 (3) Å
*b* = 11.911 (4) Å
*c* = 17.923 (6) Å
*V* = 1974.0 (11) Å^3^
*Z* = 4
Mo *K*α radiation
μ = 0.09 mm^−1^
*T* = 296 K
0.25 × 0.23 × 0.22 mm

#### Data collection

Bruker APEXII CCD diffractometer
Absorption correction: multi-scan (*SADABS*; Sheldrick, 1996 ▶) *T*_min_ = 0.977, *T*_max_ = 0.980
8939 measured reflections
3605 independent reflections
2280 reflections with *I* > 2σ(*I*)
*R*_int_ = 0.033

#### Refinement

*R*[*F* ^2^ > 2σ(*F* ^2^)] = 0.040
*wR*(*F* ^2^) = 0.111
*S* = 1.01
3605 reflections
264 parameters
H-atom parameters constrained
Δρ_max_ = 0.11 e Å^−3^
Δρ_min_ = −0.12 e Å^−3^

### 

Data collection: *APEX2* (Bruker, 2009 ▶); cell refinement: *SAINT* (Bruker, 2009 ▶); data reduction: *SAINT*; program(s) used to solve structure: *SHELXS97* (Sheldrick, 2008 ▶); program(s) used to refine structure: *SHELXL97* (Sheldrick, 2008 ▶); molecular graphics: *SHELXTL* (Sheldrick, 2008 ▶); software used to prepare material for publication: *SHELXL97*.

## Supplementary Material

Crystal structure: contains datablock(s) global, I. DOI: 10.1107/S1600536812039360/zq2182sup1.cif

Structure factors: contains datablock(s) I. DOI: 10.1107/S1600536812039360/zq2182Isup2.hkl

Supplementary material file. DOI: 10.1107/S1600536812039360/zq2182Isup3.cml

Additional supplementary materials:  crystallographic information; 3D view; checkCIF report

## Figures and Tables

**Table 1:** Hydrogen-bond geometry (Å, °)

*D*—H⋯*A*	*D*—H	H⋯*A*	*D*⋯*A*	*D*—H⋯*A*
C12—H12⋯O1^i^	0.93	2.55	3.456 (4)	165

Symmetry code: (i) 
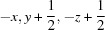
.

**Fig. 1.**
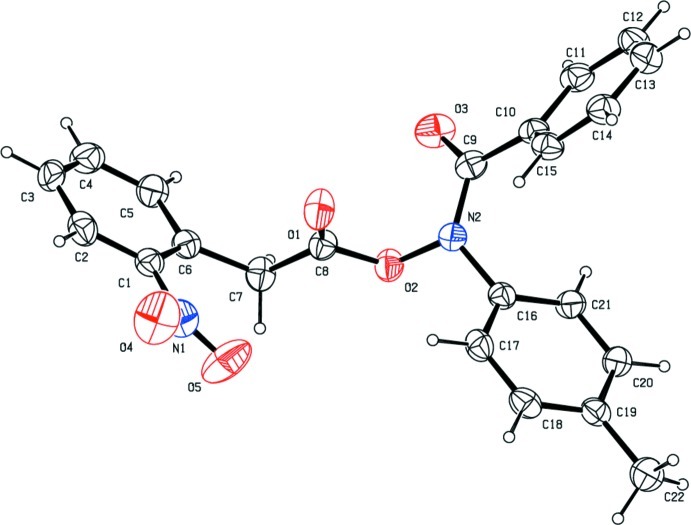
The molecular structure of the title compound with the atomic numbering and 50% probability displacement ellipsoids (H atoms are shown as small spheres of arbitrary radius).

## supplementary crystallographic information

### Comment

Hydroxamic acid derivatives have received considerable attention in recent years as the result of the discovery of their role in the biochemical toxicology of many drugs and other chemicals. Thus, these compounds continue to attract much attention as potential biological agents (Noh *et al.*, 2009; Zeng *et al.*, 2003).

The title compound, C_22_H_18_N_2_O_5_, was prepared according to the method described by Ayyangark *et al.* (1986). The molecule contains three branched chains with its centre placed at midpoint of the N2 atom (Fig. 1). The nitro-substituted benzene ring makes a dihedral angle of 71.56 (1)° with the benzoyl ring and 16.28 (1)° with the methyl-substituted benzene ring. The crystal structure features weak intra- and inter-molecular C—H···O interactions (Table 1).

### Experimental

The title compound was prepared according to the method described by Ayyangark *et al.* (1986). The yellow crystals were grown from a solution of dichloromethane–methanol (1:3 *v*/*v*) by slow evaporation at room temperature.

### Refinement

All H atoms were positioned geometrically and allowed to ride on their parent atoms, with C—H = 0.93 Å and *U*_iso_ = 1.2*U*_eq_(C) for aromatic H atoms, with C—H = 0.97 Å and *U*_iso_ = 1.2*U*_eq_(C) for methylene H atoms, and with C—H = 0.96 Å and *U*_iso_ = 1.5*U*_eq_(C) for methyl H atoms.

### Crystal data

**Table:**

C_22_H_18_N_2_O_5_	*F*(000) = 816
*M**_r_* = 390.38	*D*_x_ = 1.314 Mg m^−^^3^
Orthorhombic, *P*2_1_2_1_2_1_	Mo *K*α radiation, λ = 0.71073 Å
Hall symbol: P 2ac 2ab	Cell parameters from 2167 reflections
*a* = 9.246 (3) Å	θ = 2.5–20.3°
*b* = 11.911 (4) Å	µ = 0.09 mm^−^^1^
*c* = 17.923 (6) Å	*T* = 296 K
*V* = 1974.0 (11) Å^3^	Block, yellow
*Z* = 4	0.25 × 0.23 × 0.22 mm

### Data collection

**Table:**

Bruker APEXII CCD diffractometer	3605 independent reflections
Radiation source: fine-focus sealed tube	2280 reflections with *I* > 2σ(*I*)
Graphite monochromator	*R*_int_ = 0.033
φ and ω scans	θ_max_ = 25.5°, θ_min_ = 2.5°
Absorption correction: multi-scan (*SADABS*; Sheldrick, 1996)	*h* = −9→11
*T*_min_ = 0.977, *T*_max_ = 0.980	*k* = −14→14
8939 measured reflections	*l* = −18→21

### Refinement

**Table:**

Refinement on *F*^2^	H-atom parameters constrained
Least-squares matrix: full	*w* = 1/[σ^2^(*F*_o_^2^) + (0.0508*P*)^2^ + 0.0436*P*] where *P* = (*F*_o_^2^ + 2*F*_c_^2^)/3
*R*[*F*^2^ > 2σ(*F*^2^)] = 0.040	(Δ/σ)_max_ = 0.001
*wR*(*F*^2^) = 0.111	Δρ_max_ = 0.11 e Å^−^^3^
*S* = 1.01	Δρ_min_ = −0.12 e Å^−^^3^
3605 reflections	Extinction correction: *SHELXL97* (Sheldrick, 2008)
264 parameters	Extinction coefficient: 0.0097 (13)
0 restraints	

### Special details

**Table:**

Geometry. All e.s.d.'s (except the e.s.d. in the dihedral angle between two l.s. planes) are estimated using the full covariance matrix. The cell e.s.d.'s are taken into account individually in the estimation of e.s.d.'s in distances, angles and torsion angles; correlations between e.s.d.'s in cell parameters are only used when they are defined by crystal symmetry. An approximate (isotropic) treatment of cell e.s.d.'s is used for estimating e.s.d.'s involving l.s. planes.
Refinement. Refinement of *F*^2^ against ALL reflections. The weighted *R*-factor *wR* and goodness of fit *S* are based on *F*^2^, conventional *R*-factors *R* are based on *F*, with *F* set to zero for negative *F*^2^. The threshold expression of *F*^2^ > σ(*F*^2^) is used only for calculating *R*-factors(gt) *etc*. and is not relevant to the choice of reflections for refinement. *R*-factors based on *F*^2^ are statistically about twice as large as those based on *F*, and *R*-factors based on ALL data will be even larger.

### Fractional atomic coordinates and isotropic or equivalent isotropic displacement parameters (Å^2^)

**Table:**

	*x*	*y*	*z*	*U*_iso_*/*U*_eq_	
C1	0.3532 (3)	0.1353 (2)	−0.07101 (14)	0.0662 (7)	
C2	0.2883 (3)	0.1027 (3)	−0.13739 (17)	0.0852 (9)	
H2	0.2795	0.0271	−0.1496	0.102*	
C3	0.2368 (3)	0.1853 (4)	−0.18535 (16)	0.0961 (11)	
H3	0.1933	0.1658	−0.2303	0.115*	
C4	0.2508 (4)	0.2945 (3)	−0.16565 (19)	0.0956 (10)	
H4	0.2173	0.3502	−0.1977	0.115*	
C5	0.3124 (3)	0.3237 (3)	−0.10045 (17)	0.0817 (8)	
H5	0.3186	0.3995	−0.0886	0.098*	
C6	0.3665 (3)	0.2469 (2)	−0.05063 (13)	0.0637 (7)	
C7	0.4263 (3)	0.2894 (2)	0.02163 (14)	0.0747 (8)	
H7A	0.5196	0.2548	0.0306	0.090*	
H7B	0.4409	0.3698	0.0181	0.090*	
C8	0.3285 (3)	0.2649 (2)	0.08568 (14)	0.0648 (7)	
C9	0.1744 (3)	0.3805 (2)	0.21269 (15)	0.0675 (7)	
C10	0.0711 (3)	0.3651 (2)	0.27535 (14)	0.0613 (7)	
C11	−0.0023 (3)	0.4586 (2)	0.30155 (17)	0.0779 (8)	
H11	0.0173	0.5291	0.2817	0.094*	
C12	−0.1047 (4)	0.4469 (3)	0.35739 (19)	0.0914 (10)	
H12	−0.1526	0.5098	0.3757	0.110*	
C13	−0.1356 (4)	0.3429 (3)	0.38569 (17)	0.0914 (9)	
H13	−0.2047	0.3353	0.4230	0.110*	
C14	−0.0659 (3)	0.2509 (3)	0.35956 (16)	0.0808 (8)	
H14	−0.0882	0.1804	0.3786	0.097*	
C15	0.0376 (3)	0.2615 (2)	0.30509 (15)	0.0687 (7)	
H15	0.0858	0.1980	0.2880	0.082*	
C16	0.3668 (3)	0.2615 (2)	0.27241 (13)	0.0587 (6)	
C17	0.4158 (3)	0.1531 (2)	0.26885 (16)	0.0714 (7)	
H17	0.3964	0.1095	0.2270	0.086*	
C18	0.4936 (3)	0.1090 (2)	0.32708 (16)	0.0750 (8)	
H18	0.5281	0.0358	0.3236	0.090*	
C19	0.5220 (3)	0.1707 (2)	0.39074 (14)	0.0668 (7)	
C20	0.4722 (3)	0.2792 (2)	0.39293 (15)	0.0727 (8)	
H20	0.4898	0.3226	0.4351	0.087*	
C21	0.3968 (3)	0.3255 (2)	0.33402 (15)	0.0680 (7)	
H21	0.3663	0.3998	0.3361	0.082*	
C22	0.6051 (4)	0.1213 (3)	0.45524 (15)	0.0997 (11)	
H22A	0.5407	0.0785	0.4862	0.150*	
H22B	0.6475	0.1808	0.4841	0.150*	
H22C	0.6802	0.0733	0.4365	0.150*	
N1	0.4064 (4)	0.0451 (2)	−0.02206 (17)	0.0970 (8)	
N2	0.2881 (2)	0.3064 (2)	0.21067 (12)	0.0713 (6)	
O1	0.2230 (3)	0.20874 (17)	0.08620 (10)	0.0923 (7)	
O2	0.37898 (19)	0.32093 (15)	0.14693 (9)	0.0750 (5)	
O3	0.1555 (2)	0.44895 (17)	0.16349 (12)	0.0973 (7)	
O4	0.3608 (4)	−0.0472 (2)	−0.03322 (16)	0.1591 (12)	
O5	0.4911 (4)	0.0655 (2)	0.02581 (17)	0.1589 (13)	

### Atomic displacement parameters (Å^2^)

**Table:**

	*U*^11^	*U*^22^	*U*^33^	*U*^12^	*U*^13^	*U*^23^
C1	0.0596 (16)	0.0736 (17)	0.0655 (17)	0.0017 (14)	0.0069 (14)	0.0037 (14)
C2	0.072 (2)	0.104 (2)	0.080 (2)	−0.0155 (18)	0.0139 (17)	−0.0225 (19)
C3	0.0593 (19)	0.175 (4)	0.0541 (18)	0.000 (2)	0.0040 (15)	−0.003 (2)
C4	0.080 (2)	0.123 (3)	0.084 (2)	0.017 (2)	0.0039 (19)	0.026 (2)
C5	0.085 (2)	0.0853 (19)	0.0753 (19)	0.0026 (17)	0.0079 (17)	0.0145 (17)
C6	0.0573 (16)	0.0715 (17)	0.0622 (15)	−0.0049 (14)	0.0097 (13)	0.0099 (14)
C7	0.078 (2)	0.0780 (17)	0.0681 (17)	−0.0196 (15)	0.0065 (16)	0.0010 (14)
C8	0.0636 (18)	0.0685 (16)	0.0624 (15)	−0.0117 (15)	−0.0025 (14)	0.0002 (13)
C9	0.0651 (19)	0.0595 (16)	0.0780 (18)	−0.0091 (15)	−0.0111 (15)	0.0001 (14)
C10	0.0515 (15)	0.0589 (15)	0.0735 (17)	0.0002 (13)	−0.0114 (13)	−0.0039 (13)
C11	0.0778 (19)	0.0615 (16)	0.095 (2)	0.0096 (17)	−0.0190 (19)	−0.0074 (15)
C12	0.080 (2)	0.098 (2)	0.096 (2)	0.028 (2)	−0.015 (2)	−0.037 (2)
C13	0.072 (2)	0.117 (3)	0.085 (2)	0.006 (2)	0.0023 (18)	−0.013 (2)
C14	0.0641 (19)	0.089 (2)	0.090 (2)	−0.0034 (17)	0.0018 (17)	0.0038 (18)
C15	0.0571 (18)	0.0627 (17)	0.0864 (18)	0.0001 (13)	−0.0021 (15)	−0.0073 (14)
C16	0.0491 (14)	0.0635 (15)	0.0636 (15)	−0.0052 (12)	0.0035 (13)	−0.0056 (13)
C17	0.0775 (19)	0.0649 (17)	0.0717 (17)	−0.0025 (15)	0.0056 (15)	−0.0173 (14)
C18	0.0778 (19)	0.0603 (16)	0.0869 (19)	0.0090 (16)	0.0150 (17)	0.0030 (15)
C19	0.0643 (17)	0.0671 (17)	0.0690 (17)	0.0077 (14)	0.0126 (15)	0.0068 (14)
C20	0.0708 (19)	0.0748 (18)	0.0725 (17)	0.0015 (15)	−0.0061 (16)	−0.0120 (14)
C21	0.0658 (17)	0.0614 (15)	0.0768 (18)	0.0042 (15)	−0.0112 (14)	−0.0112 (13)
C22	0.107 (3)	0.114 (2)	0.0781 (19)	0.025 (2)	0.0122 (19)	0.0285 (18)
N1	0.119 (2)	0.0711 (18)	0.101 (2)	0.0019 (18)	0.0082 (18)	0.0082 (16)
N2	0.0609 (14)	0.0913 (16)	0.0618 (13)	0.0061 (13)	−0.0007 (12)	−0.0030 (11)
O1	0.0954 (14)	0.1119 (16)	0.0698 (12)	−0.0502 (14)	0.0065 (11)	−0.0052 (11)
O2	0.0661 (12)	0.0943 (13)	0.0647 (11)	−0.0177 (11)	0.0017 (10)	−0.0068 (10)
O3	0.0899 (15)	0.0888 (13)	0.1131 (16)	−0.0055 (13)	−0.0006 (13)	0.0337 (13)
O4	0.238 (4)	0.0828 (17)	0.157 (2)	−0.020 (2)	−0.017 (3)	0.0193 (16)
O5	0.192 (3)	0.116 (2)	0.169 (3)	0.001 (2)	−0.093 (3)	0.0317 (19)

### Geometric parameters (Å, º)

**Table:**

C1—C6	1.384 (4)	C12—H12	0.9300
C1—C2	1.387 (3)	C13—C14	1.354 (4)
C1—N1	1.472 (4)	C13—H13	0.9300
C2—C3	1.390 (4)	C14—C15	1.373 (4)
C2—H2	0.9300	C14—H14	0.9300
C3—C4	1.354 (5)	C15—H15	0.9300
C3—H3	0.9300	C16—C17	1.369 (4)
C4—C5	1.346 (4)	C16—C21	1.370 (3)
C4—H4	0.9300	C16—N2	1.429 (3)
C5—C6	1.373 (4)	C17—C18	1.372 (4)
C5—H5	0.9300	C17—H17	0.9300
C6—C7	1.496 (3)	C18—C19	1.383 (4)
C7—C8	1.490 (3)	C18—H18	0.9300
C7—H7A	0.9700	C19—C20	1.372 (3)
C7—H7B	0.9700	C19—C22	1.508 (4)
C8—O1	1.183 (3)	C20—C21	1.380 (3)
C8—O2	1.367 (3)	C20—H20	0.9300
C9—O3	1.214 (3)	C21—H21	0.9300
C9—N2	1.372 (3)	C22—H22A	0.9600
C9—C10	1.486 (4)	C22—H22B	0.9600
C10—C15	1.379 (3)	C22—H22C	0.9600
C10—C11	1.386 (3)	N1—O5	1.187 (3)
C11—C12	1.385 (4)	N1—O4	1.194 (3)
C11—H11	0.9300	N2—O2	1.429 (3)
C12—C13	1.369 (4)		
			
C6—C1—C2	122.2 (3)	C14—C13—H13	119.9
C6—C1—N1	120.9 (3)	C12—C13—H13	119.9
C2—C1—N1	116.9 (3)	C13—C14—C15	120.2 (3)
C1—C2—C3	118.7 (3)	C13—C14—H14	119.9
C1—C2—H2	120.6	C15—C14—H14	119.9
C3—C2—H2	120.6	C14—C15—C10	120.9 (3)
C4—C3—C2	119.0 (3)	C14—C15—H15	119.6
C4—C3—H3	120.5	C10—C15—H15	119.6
C2—C3—H3	120.5	C17—C16—C21	119.7 (2)
C5—C4—C3	121.0 (3)	C17—C16—N2	119.1 (2)
C5—C4—H4	119.5	C21—C16—N2	121.2 (2)
C3—C4—H4	119.5	C16—C17—C18	119.9 (2)
C4—C5—C6	123.1 (3)	C16—C17—H17	120.0
C4—C5—H5	118.4	C18—C17—H17	120.0
C6—C5—H5	118.4	C17—C18—C19	121.6 (2)
C5—C6—C1	115.8 (3)	C17—C18—H18	119.2
C5—C6—C7	118.2 (3)	C19—C18—H18	119.2
C1—C6—C7	125.9 (2)	C20—C19—C18	117.4 (2)
C8—C7—C6	112.1 (2)	C20—C19—C22	121.1 (3)
C8—C7—H7A	109.2	C18—C19—C22	121.5 (2)
C6—C7—H7A	109.2	C19—C20—C21	121.6 (3)
C8—C7—H7B	109.2	C19—C20—H20	119.2
C6—C7—H7B	109.2	C21—C20—H20	119.2
H7A—C7—H7B	107.9	C16—C21—C20	119.8 (2)
O1—C8—O2	123.5 (2)	C16—C21—H21	120.1
O1—C8—C7	128.1 (2)	C20—C21—H21	120.1
O2—C8—C7	108.4 (2)	C19—C22—H22A	109.5
O3—C9—N2	121.5 (3)	C19—C22—H22B	109.5
O3—C9—C10	122.6 (3)	H22A—C22—H22B	109.5
N2—C9—C10	115.7 (2)	C19—C22—H22C	109.5
C15—C10—C11	118.6 (3)	H22A—C22—H22C	109.5
C15—C10—C9	123.1 (2)	H22B—C22—H22C	109.5
C11—C10—C9	118.1 (2)	O5—N1—O4	122.9 (3)
C12—C11—C10	119.9 (3)	O5—N1—C1	120.1 (3)
C12—C11—H11	120.1	O4—N1—C1	117.0 (3)
C10—C11—H11	120.1	C9—N2—O2	113.2 (2)
C13—C12—C11	120.1 (3)	C9—N2—C16	127.6 (2)
C13—C12—H12	119.9	O2—N2—C16	111.39 (19)
C11—C12—H12	119.9	C8—O2—N2	112.48 (19)
C14—C13—C12	120.3 (3)		
			
C6—C1—C2—C3	0.8 (4)	C21—C16—C17—C18	0.5 (4)
N1—C1—C2—C3	−179.9 (3)	N2—C16—C17—C18	179.0 (2)
C1—C2—C3—C4	−0.3 (4)	C16—C17—C18—C19	1.3 (4)
C2—C3—C4—C5	−0.6 (5)	C17—C18—C19—C20	−1.5 (4)
C3—C4—C5—C6	1.0 (5)	C17—C18—C19—C22	178.9 (3)
C4—C5—C6—C1	−0.5 (4)	C18—C19—C20—C21	0.0 (4)
C4—C5—C6—C7	−177.2 (3)	C22—C19—C20—C21	179.6 (3)
C2—C1—C6—C5	−0.4 (4)	C17—C16—C21—C20	−2.0 (4)
N1—C1—C6—C5	−179.7 (3)	N2—C16—C21—C20	179.6 (2)
C2—C1—C6—C7	176.0 (2)	C19—C20—C21—C16	1.8 (4)
N1—C1—C6—C7	−3.3 (4)	C6—C1—N1—O5	−18.3 (5)
C5—C6—C7—C8	106.5 (3)	C2—C1—N1—O5	162.4 (3)
C1—C6—C7—C8	−69.8 (4)	C6—C1—N1—O4	161.9 (3)
C6—C7—C8—O1	8.2 (4)	C2—C1—N1—O4	−17.4 (4)
C6—C7—C8—O2	−170.7 (2)	O3—C9—N2—O2	1.1 (3)
O3—C9—C10—C15	141.9 (3)	C10—C9—N2—O2	176.52 (19)
N2—C9—C10—C15	−33.4 (3)	O3—C9—N2—C16	147.4 (3)
O3—C9—C10—C11	−33.6 (4)	C10—C9—N2—C16	−37.2 (3)
N2—C9—C10—C11	151.1 (2)	C17—C16—N2—C9	144.5 (3)
C15—C10—C11—C12	1.2 (4)	C21—C16—N2—C9	−37.0 (4)
C9—C10—C11—C12	176.8 (2)	C17—C16—N2—O2	−68.7 (3)
C10—C11—C12—C13	−1.3 (4)	C21—C16—N2—O2	109.7 (3)
C11—C12—C13—C14	0.3 (5)	O1—C8—O2—N2	−1.6 (4)
C12—C13—C14—C15	0.8 (4)	C7—C8—O2—N2	177.4 (2)
C13—C14—C15—C10	−0.9 (4)	C9—N2—O2—C8	−84.8 (2)
C11—C10—C15—C14	−0.1 (4)	C16—N2—O2—C8	123.4 (2)
C9—C10—C15—C14	−175.5 (2)		

### Hydrogen-bond geometry (Å, º)

**Table:**

*D*—H···*A*	*D*—H	H···*A*	*D*···*A*	*D*—H···*A*
C7—H7*A*···O5	0.97	2.27	2.734 (4)	108
C12—H12···O1^i^	0.93	2.55	3.456 (4)	165

Symmetry code: (i) −*x*, *y*+1/2, −*z*+1/2.
